# Effects of Weaning Age on Plasma Biomarkers and Growth Performance in Simmental Calves

**DOI:** 10.3390/ani12091168

**Published:** 2022-05-02

**Authors:** Giulia Ferronato, Luca Cattaneo, Erminio Trevisi, Luigi Liotta, Andrea Minuti, Francesca Arfuso, Vincenzo Lopreiato

**Affiliations:** 1 Department of Civil Engineering, Architecture, Environment, Land Planning and Mathematics (DICATAM), Università degli Studi di Brescia, 25121 Brescia, Italy; giulia.ferronato@unibs.it; 2 Department of Animal Sciences, Food and Nutrition, Faculty of Agriculture, Food and Environmental Science, Università Cattolica del Sacro Cuore, 29122 Piacenza, Italy; luca.cattaneo@unicatt.it (L.C.); erminio.trevisi@unicatt.it (E.T.); andrea.minuti@unicatt.it (A.M.); 3 Department of Veterinary Sciences, Università di Messina, 98168 Messina, Italy; luigi.liotta@unime.it (L.L.); francesca.arfuso@unime.it (F.A.)

**Keywords:** calves, calf management, early weaning, metabolic profile, plasma biomarkers

## Abstract

**Simple Summary:**

Weaning plays a vital role in the management strategy of a dairy farms, also affecting the future animals’ performances, such as growth, reproduction, and lactation. At the same time weaning strategy, as days at weaning, influences the feeding cost as milk supply during the pre-weaning period reaches 40% of the total feed costs. The aim of the study was to investigate the effects of weaning age, conventional (60 days) or early (45 days) on growth performances and inflammometabolic status of ten Simmental calves. The proposed results showed how early weaning strategy seemed to not affect inflammatory status and liver functionality after weaning, highlighting the possibility to reduce rearing costs but not jeopardizing calf development, as long as calves can reach body gains as reported in the present study. At least for performance, we are aware that the low number of calves enrolled in each treatment can limit the recommendations based on our findings. Hence, this study should be considered more like an explorative investigation since no recent data are available for Simmental calves in terms of growth performance and inflammometabolic adaptation.

**Abstract:**

Weaning plays a key role in health status and future performance of calves. The aim of this study was to investigate the effects of weaning age (Wa), early (45 d, EW) or conventional (60 d, CW), on growth performance and metabolic profile of ten Simmental calves (5 EW and 5 CW calves). Daily intake of milk and calf starter was recorded. Blood samples and measurements of body weight (BW), heart girth (HG), and wither height (WH) were collected at −25, −15, 0, 6, and 20 days relative to weaning. Growth performances (BW, HG, WH) were affected by Wa, resulting lower in EW calves compared with CW calves (*p* < 0.05). Average daily gain was affected by overall Wa and Time but also by the interaction Wa × Time (*p* < 0.05). EW calves had lower paraoxonase and higher oxidation protein products levels, lower glucose levels in the post-weaning period, lower Ca and cholesterol levels at 20 d after weaning, and higher GGT activity at −25 d from weaning (*p* < 0.05). A significant interaction effect between Wa and Time was reached for glucose, Ca, cholesterol. In conclusion, weaning Simmental calves at approximately six weeks of age might not affect inflammatory status and liver functionality after weaning. As secondary outcome, even though the low number of animals could represent a limitation, the average daily gain obtained by Simmental calves weaned at 45 d supported this strategy (despite the lower body weight at weaning and after was due only to the age difference of 15 days). Hence, in order to reduce rearing costs, early weaning for Simmental calves (dual-purpose breed, milk and beef) might not jeopardize calf development, as long as calves can reach body gains as reported in the present study.

## 1. Introduction

Weaning in mammals is a crucial and stressful time as it involves social, environmental, and feeding changes. Dairy calves are usually separated from the dam after calving to better control health conditions and the colostrum and feeding intake, even if they are fed milk artificially until weaning. Considering the impact of milk supply on calf well-being, behavior, and health status [1], several hypotheses of weaning management best practices have been investigated to determine the optimal weaning age and to evaluate the most appropriate feeding strategy [2,3,4,5]. According to Eckert et al. [6], Holstein calves fed a higher quantity of milk pre-weaning (1.2 kg/d of MR) have more nutrient intake, higher growth rates, more gastrointestinal development at weaning when weaned at eight weeks compared with calves weaned at six weeks of age.

From a physiology perspective, it is well known that milk intake, in terms of quantity and timing, affects rumen physiology and morphology. In particular, prolonged milk supply may slow the rumen and rumen papillae development with consequences on solid feed intake capacity, VFA profile, and composition of the ruminal and intestinal microbiomes [7,8]. Conversely, a restricted milk supply and stressful weaning transition may affect the calf behavior and growth performance [9].

Considering the high feed cost related to milk supply in the pre-weaning phase, reaching 40% of total feed costs [6], early weaning strategy has been investigated as a way to reduce milk feeding days [10,11]. In a previous study, Lopreiato et al. [12] showed that early weaning age (45 d versus 60 d) did not affect rumen behavior and development during the post-weaning phase, reducing, in turn, rearing costs.

Simmental cattle, a dual purpose worldwide breed common in central Europe, is becoming attractive and economic to particularly smallholders that produce milk to sell or to produce cheese by themselves. Its breeding program aims to improve both dairy and beef traits, and an economic selection index has been developed for this purpose [13]. Thus, Simmental farmers can couple a reasonable milk yield and a higher profit given from selling bulls both at young and adult age after fattening.

To the best of our knowledge, no data are available reporting performance and metabolic adaptation of Simmental calves reared under a dairy system. Therefore, the present study aimed to investigate the effect of age at weaning on plasma metabolic parameters and growth performance, in terms of body weight (BW), heart girth (HG), and wither height (WH) in Simmental calves reared under a conventional dairy system.

## 2. Materials and Methods

### 2.1. Calves Management and Experimental Design

Ten Simmental calves were enrolled. Within 4 h from birth, calves were weighed and then transferred to individual straw-bedded pens (2.8 m long × 1.80 m wide). According to birth body weight (BW) and sex, each calf was randomly assigned to one of two weaning protocols: 5 calves (3 males and 2 females) were weaned at 45 d (EW) and 5 calves (3 males and 2 females) were weaned at 60 d (CW). All calves received 3 L of their dam’s colostrum by nipple bottle. If voluntary colostrum intake did not reach the required 3 L, the remaining colostrum quantity was administrated with an esophageal feeder (Speedy Drencher XL, Agri-Zoo San Marino SRL, Domagnano, San Marino). Colostrum quality was measured with a Brix refractometer (HHTEC, Hong Han GmbH, Heidelberg, Germany). If it was measured less than 25% on the Brix scale, frozen colostrum of sufficient quality was thawed and fed to the calf. After the first colostrum feeding, animals received four meals of their dam’s transition milk over 2 days (at 12, 24, 36, and 48 h after birth). The EW group received 6 L of bulk whole milk from d 3 to 38, stepped down to 3 L/d on d 39, and weaned on d 45. The CW group received 6 L of bulk whole milk from d 3 to 53, stepped down to 3 L/d on d 54, and weaned on d 60. When calves were receiving 6 L of milk, meals were delivered twice at 0530 and 1800 h, whereas during the step-down week, calves received milk only once at 0530 h. From birth, pelleted calf starter (Dietovit Excellence, SIVAM Spa, Casalpusterlengo, Lodi, Italy; Table 1) was offered daily ad libitum at 0700 h. The initial daily amount of starter offered was 0.1 kg, increasing by 0.2 kg as intake increased. After weaning, for both groups, grass hay was offered ad libitum until the end of the experiment (3 weeks after weaning), refilling once daily in the morning. Composition of bulk whole milk, calf starter, and grass hay are shown in Table 1.

### 2.2. Body Measurements, Blood Samples, and Analysis

BW, HG, and WH of calves were recorded at birth and then at −25, −15, 0, 6, and 20 d relative to weaning at 0600 ± 0030 h, before milk and starter distribution.

Blood samples were collected from the jugular vein using 9-mL vacutainers test tubes with lithium heparin (Vacutest Kima srl, Arzergrande, PD, Italy) at −25, −15, 0, 6, and 20 d relative to weaning, in the morning before milk and starter distribution. After collection, blood samples were immediately cooled in an ice-water bath and then centrifuged at 1900× *g* for 16 min at 4 °C. Plasma was aliquoted and stored at −20 °C until metabolites analysis. Samples of plasma were analyzed at 37 °C by an automated clinical analyzer (ILAB 650, Instrumentation Laboratory Company, Lexington, MA, USA). Commercial kits were used to measure glucose, total cholesterol, urea, calcium, inorganic phosphorus, magnesium, total protein, albumin, total bilirubin, and creatinine (Instrumentation Laboratory SpA, Milan, Italy), non-esterified fatty acids (NEFA), zinc (Wako, Chemicals GmbH, Neuss, Germany), and β-hydroxybutyric acid (BHB, kit Ranbut, Randox Laboratories Ltd., Crumlin, Co Antrim, United Kingdom). Electrolytes (Ca, Na, K, and Cl) were detected by the potentiometer method (Ion Selective Electrode connected to ILAB 650). Kinetic analysis was adopted to determine the activity of enzymes: aspartate aminotransferase (AST, EC 2.6.1.1) and γ-glutamyltransferase (GGT, EC 2.3.2.2) using commercial kits (Instrumentation Laboratory SpA, Milan, Italy). The total globulin fraction was determined by subtracting the albumin from the total protein. Haptoglobin and ceruloplasmin concentrations were measured using methods proposed by Bertoni et al. [14] adapted to ILAB 650 conditions. Plasma paraoxonase (PON, 3.1.8.1) activity was assessed by adapting the method of Ferré et al. [15] to the ILAB 650. Oxidative stress was evaluated measuring the total reactive oxygen metabolites (ROMt), and myeloperoxidase (MPO, EC 1.11.2.2) as described by Bionaz et al. [16]; ferric reducing antioxidant power (FRAP) using the colorimetric method and advanced oxidation protein products (AOPP) as described by Lopreiato et al. [17].

### 2.3. Statistical Analysis

All statistical analyses were performed using the SAS software package (version 9.3; SAS Inst. Inc., Cary, NC, USA). Data were tested for normality with the Shapiro–Wilk test (SAS Inst. Inc.). Normalized data were subjected to a mixed model for repeated measures (MIXED procedure; SAS Inst. Inc.) including as fixed effect the weaning age (Wa; 45 or 60 d), the days relative to weaning (Time), and their interaction (Wa × Time). Calves were included in the model as a random effect. The MIXED procedure of SAS was performed using the following covariance structures: Autoregressive (1), Compound Symmetry, Spatial Power, Unstructured, Toeplitz, and Variance Components. The covariance structure with the lowest value of Akaike’s information criterion was chosen for each variable. Statistical significance was declared at *p* < 0.05, whereas tendencies were considered at 0.05 < *p* < 0.10. In addition, Bonferroni’s post hoc procedure was used to compare least squares means between groups at each time point.

## 3. Results

The proposed results are referred to the days from weaning. Day 0 (the day of weaning) corresponds to 45 and 60 days of life, respectively, for EW and CW calves.

### 3.1. Growth Performance

**Figure 1:**
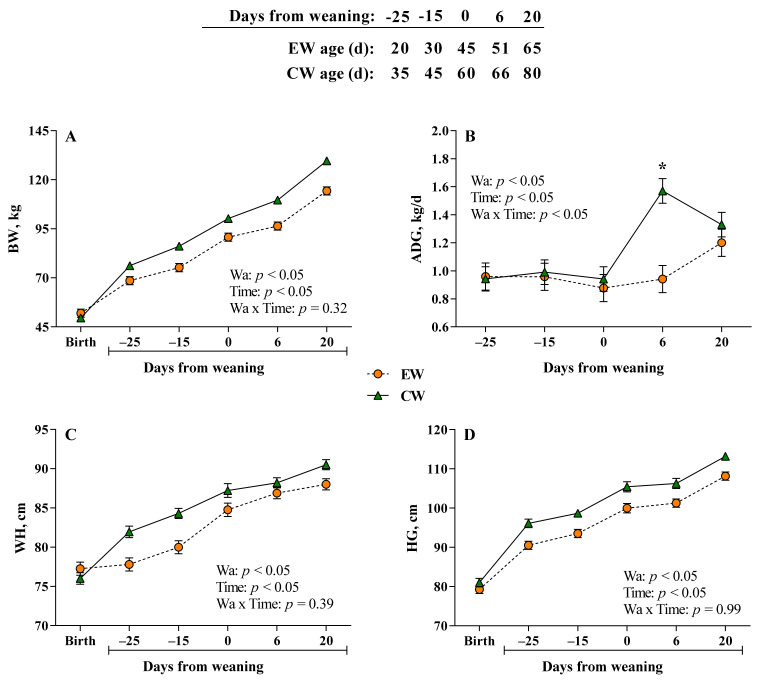
Body weight (BW; (**A**)), average daily gain (ADG; (**B**)), wither height (WH; (**C**)), and heart girth (HG; (**D**)) at −25, −15, 0, 6, and 20 d relative to weaning of Simmental calves weaned at 45 (EW) or 60 (CW) d of age. Error bars indicate the standard error of the mean (SEM). Asterisks (*) represents differences at *p* < 0.05 within each time point.

**Figure 2:**
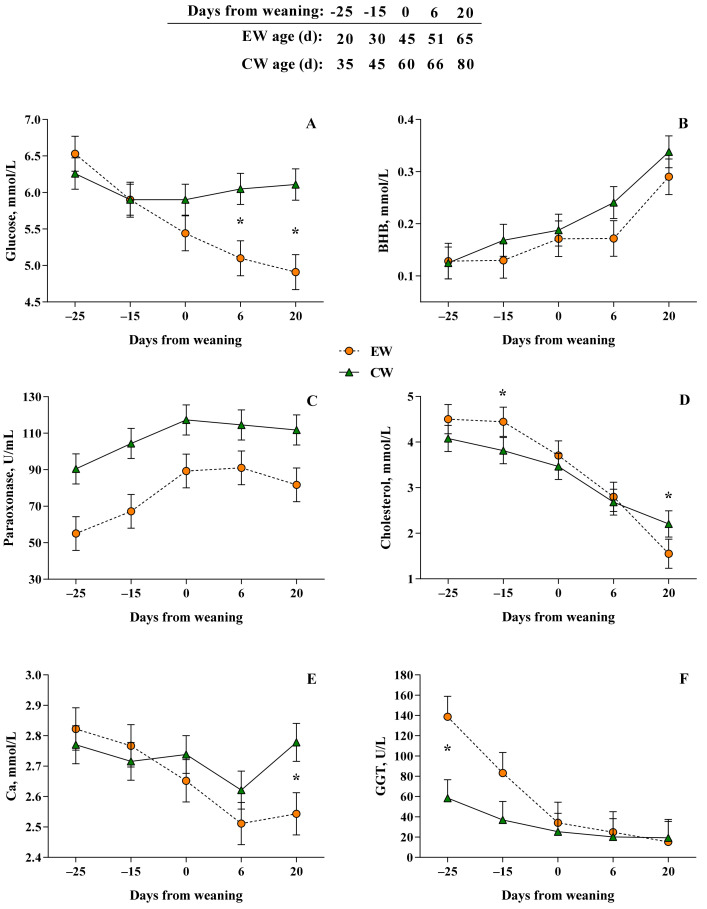
Plasma concentrations of glucose (**A**), BHB (**B**), paraoxonase (PON; (**C**)), cholesterol (**D**), calcium (**E**), and GGT (**F**) at −25, −15, 0, 6, and 20 d relative to weaning of Simmental calves weaned at 45 (EW) or 60 (CW) d of age. Error bars indicate the standard error of the mean (SEM). Asterisks (*) represents differences at *p* < 0.05 within each time point.

On average, calves between groups had similar BW at birth (51.97 ± 2.53 kg vs. 49.50 ± 2.09 kg, respectively, for EW and CW calves). As expected, calves weaned at 60 d (CW) resulted in an overall greater BW compared with EW calves (*p* < 0.05). The average BW at weaning and after 20 d was 90.7 ± 0.97 and 114.3 ± 2.10 kg for EW calves, respectively, whereas for CW was 100.2 ± 1.87 and 129.6 ± 1.87 (Figure 1A). Overall, EW calves had lower ADG compared with CW calves (0.98 ± 0.04 vs. 1.15 ± 0.03 kg/d; Wa: *p* < 0.05) due to the relevant difference obtained during the week after weaning (effect of the interaction Wa × Time: *p* < 0.05), where CW calves achieved gains of 1.57 ± 0.09 kg/d against 0.95 ± 0.1 kg/d of EW calves (*p* < 0.05; Figure 2B). However, during the period between 6 and 20 d after weaning, ADG did not differ between groups (1.33 ± 0.08 vs. 1.20 ± 0.09 kg/d for CW and EW, respectively; *p* > 0.05). Overall, WH and HG were lower in EW group compared with CW (*p* < 0.05; Figure 1C,D, respectively). Moreover, WH and HG increased over time (*p* < 0.05). Data about milk, calf starter, and hay intake were already published previously [12]. Briefly, no differences in milk intake were observed, whereas differences in starter intake between groups were achieved after weaning, where during the last four days (17, 18, 19, and 20 days after weaning), CW calves resulted in greater intake compared with EW calves [12].

### 3.2. Plasma Biomarkers

Regardless of weaning age effect, levels of NEFA, triglycerides, ceruloplasmin, ROM, and albumin were affected by Time, showing in both groups a decrease from pre- through post-weaning period (*p* ≤ 0.05; Table 2, Table 3 and Table 4). Plasma urea, Zn, P, creatinine, total protein, globulin, haptoglobin, AST, MPO, and FRAP were not affected either by weaning age, time, or their interaction (*p* > 0.05; Table 2, Table 3 and Table 4).

Overall, plasma concentrations of glucose and BHB tended to be lower in EW calves compared with CW calves throughout the entire period monitored (Wa: *p* = 0.06 and *p* = 0.09, respectively; Table 2). Specifically, a weaning age by time interaction (Wa × Time: *p* < 0.05) was observed for glucose (Figure 2A), where EW calves at 6 and 20 d (5.10 and 4.91 ± 0.23 mmol/L, respectively) had lower levels compared with CW calves (6.05 and 6.11 ± 0.21 mmol/L, respectively). It is noteworthy to report that BHB levels in both groups showed an increasing trend from the pre- through the post-weaning period (Time: *p* < 0.05; Figure 2B).

Overall, EW calves showed greater levels of AOPP (71.11 ± 4.79 vs. 56.85 ± 4.29 μmol/L, respectively, for EW and CW calves; Wa: *p* < 0.05; Table 3) and lower levels of PON (76.85 ± 7.9 vs. 107.7 ± 7.1 U/mL, respectively, for EW and CW calves; Wa: *p* < 0.05; Table 4) compared with CW calves. In particular, the transition from liquid to solid feeds and the aging of calves led to an overall decrease of AOPP levels (Time: *p* < 0.05; Table 3) and an overall increase of PON activity (Time: *p* < 0.05; Table 4 and Figure 2C).

The effect of the interaction Wa × Time was observed for Ca and cholesterol levels (*p* < 0.05). In detail, EW calves had lower levels of these two plasma biomarkers three weeks after weaning compared with CW calves (Wa × Time: *p* < 0.05; Figure 2D,E, respectively, for Ca and cholesterol). Values reached three weeks after weaning by EW and CW for Ca concentrations were 2.54 ± 0.07 and 2.78 ± 0.06 mmol/L, respectively, while for cholesterol concentrations were 1.55 ± 0.32 and 2.20 ± 0.28, respectively.

EW calves had higher activity of GGT compared with CW calves at −25 d from weaning (138.70 ± 20.23 and 58.53 ± 18.09 U/L, respectively; Wa × Time: *p* < 0.05). However, over time, GGT activity gradually decreased in both groups (Time: *p* < 0.05; Figure 2F). ALP was significantly (*p* < 0.05) affected only by Time with decreasing trend and lowest values during the post-weaning phase (Table 4).

## 4. Discussion

Understanding the impact of weaning age on the health status and growth of young ruminants is crucial in order to provide a successful optimization of future animal performance. Usually, calves are weaned between the 8th and 10th weeks of life [12,18]. Little is known concerning the metabolic response of calves to milk removal and weaning stress. This opens a great interest for researchers and farmers to better understand which are the metabolic and performance consequences immediately after weaning if early weaning is considered. Traditionally, early weaning has been recommended to accelerate rumen development, eventually reducing the cost to feed a calf [19,20]. Thus, the present study aimed to evaluate the effect of weaning age in Simmental calves (cattle breed selected for both meat and milk production) under a dairy system management between “early” weaning (45 days of life) and “conventional” weaning (60 days of life) on growth performance and metabolic response.

Although the methodological approach applied in the current study cannot describe differences purely associated with calves’ age, the aim of our investigation was to evaluate the effect of weaning at different age of calves. The benefit of our way to analyze the current data relies on an accurate assessment of the response of variables after weaning, highlighting at the same time how these variables were before weaning. It is noteworthy, however, that before weaning, the differences observed between groups depended only on the age of calves.

Although growth performance (BW, HG, WH) of EW calves were clearly lower than CW calves due to the younger age (15 days younger), calves weaned at 45 d still achieved adequate growth performance by the end of the trial [21]. The latter was firmly supported by ADG values achieved by EW calves, being overall similar with those of CW calves in the pre-weaning period and then still not different at 20 d after weaning. On the basis of starter intake data, already previously published [12], calves weaned at 45 d of age consumed similar amounts of starter compared with calves weaned at 60 d of age during both pre-weaning and step-down periods. Thus, when a limited amount of milk is fed during the weaning phase (reduction of milk at 50%), calves compensate for the milk reduction increasing starter intake. However, the amount of starter intake reached by EW calves at weaning time is worth mentioning, resulting in more than 0.9 kg/d for several consecutive days. Thereby, calves weaned at 45 d achieved optimal starter intake during step-down, weaning, and post-weaning periods, which led to a longstanding effect on rumen development [12], and growth (based on ADG recorded herein).

Numerous studies have pointed out firmly that preweaning high plane of nutrition (high milk replacer amount and high protein content of milk replacer) leads to a large growth depression if weaning occurs before eight weeks of age [6,22,23]. According to Rosadiuk et al. [24], calves of the current study were fed under a low/moderate plane of nutrition, where calves received only 6 L/d of whole milk during the entire pre-weaning period. It was previously reported that a low amount of whole milk delivered to calves before weaning (5 L/d) has led calves to consume more starter than calves fed a higher amount of whole milk (10 L/d) during the entire pre-weaning period, reaching an average of more than 0.9 kg/d of starter intake around weaning [24]. Based on data from the present and Rosadiuk et al. [24] studies, feeding calves with whole milk (approximately 30–35% of fat and 25–30% of crude protein on DM basis) at a rate of 5–6 L/d would not have negative implication on growth and solid intake during the pre- and post-weaning period. However, from the data herein obtained it should be highlighted that a certain effect of breed could account for growth performance when comparing with Holstein calves. Even though it was not our purpose, looking at intakes and growth rate of Holstein calves from previous studies with similar or higher amount of whole milk or milk replacer, Simmental calves of the current trial had on average a greater efficiency considering the starter and milk consumed. For instance, comparing our data with data from the Rosadiuk et al. [24] study, Holstein calves that received 5 L/d of whole milk had a lower ADG, regardless of the time of weaning. When the effect of weaning age is considered, Simmental calves weaned early showed a higher ADG at the similar time points compared with Holstein calves weaned at six weeks in the study of Eckert et al. [6], where calves received a relevant greater amount of milk (as milk replacer, 1.2 kg/d) and had a relatively greater intake of starter from approximately 20 d through 57 d of age. These differences in growth rate are of great relevance because they point out a certain degree of diversity in feed efficiency between calves of a dual-purpose breed (Simmental) and Holstein calves under a dairy system management. However, only a specific trial accounting for the breed effect under the same management could give valuable outcomes.

Among biomarkers associated with energy metabolism, glucose can be considered the primary energy source for pre-weaning calves and it is linked with the milk supply [25]. In calves, glucose is the most affected plasma biomarker during ruminal development, due to the shift from a glycolytic to a glucogenic liver [26] that causes a decreased blood glucose concentration. According to Haga et al. [27] and Lopreiato et al. [3], when calves increase starter intake, the rumen in turn develops, resulting in a decrease in intestinal absorption of dietary glucose. Hence, most of the plasma glucose, especially after weaning, is derived from hepatic gluconeogenesis starting from ruminal propionate [28]. Thus, the lower glucose concentration in EW calves during and after weaning could likely reflect a reduced starter feed intake and rumen papillae development, which in turn might reflect a lower VFA absorption on an equal liver metabolic activity. The general decrease of glucose occurred simultaneously with BHB increase, an indicator of development in pre-ruminant calves [9,29]. As reported above, weaning marks the transition from gut to rumen as a site of production and/or absorption of energy sources [26]. As the rumen becomes functional, increasing the activity of microbial fermentation in calves, production of VFA increases as well, leading to greater ketogenesis from butyrate in the rumen epithelial metabolism. Then, BHB resulting from the oxidation of butyrate enters the bloodstream [26]. Regardless of weaning age, the rapid increase of BHB in post weaning phase is consistent with previous studies [6,30] and likely demonstrates the increasing ketogenic capacity of the rumen epithelium with age and greater amount of concentrates. The lack of differences between EW and CW calves during the post-weaning phase may be attributed to an efficient rumen development and a satisfactory VFA production as reported by Lopreiato et al. [12]. Based on BHB data, we may speculate that EW calves in the present study were characterized by a metabolic function of the rumen wall and an efficiency in converting butyrate to BHB similar to calves weaned 15 days later.

The effect of weaning age on liver function, inflammatory response, and oxidative stress was also assessed throughout the experimental period. Weaning is a multifaced stressor that usually involves several husbandry practices, including the complete removal of milk at a very young age, nutritional adjustment to a non-milk diet, and social reorganization. The potential alteration of the immune and inflammatory state and the metabolic activity of liver occur through mechanisms of physiological adaptation in response to disrupted homeostasis. Thus, the “homeoretic” systemic reaction to this stress encompasses a wide range of immunological and inflammatory responses, but also involves the liver, due to the drastic shift in nutrient utilization. Regarding the inflammatory status, ceruloplasmin, one of the main acute phase protein biomarkers, was only time-dependent, decreasing with age and showing that both groups had a controlled physiological response to weaning adaptation and gut microbiota colonization [31]. This was confirmed also by cholesterol that, in calves, depends on the amount of fat absorbed from GIT and mobilized lipids that are re-esterified into low-density lipoprotein by the liver [32]. Tendentially, the values increase during milk feeding and decrease after weaning [33]. Since milk feeding exclusively provides dietary lipids during the first weeks of life, this led to the greater levels of cholesterol observed before rather than after weaning, regardless of the weaning age effect. However, the difference at 20 d after weaning between CW and EW calves, where the latter had lower levels, should be ascribed to the lower starter intake, which in turn results in a lower dietary lipid ingestion. In fact, during the last four days (17, 18, 19, and 20 days after weaning), CW calves had greater starter intake compared with EW calves: 3.98 ± 0.67 kg vs. 2.96 ± 0.74, respectively, for EW and CW calves as reported in our previous published paper [12]. On this basis, it might be excluded that EW calves had lower liver functionality due to an inflammatory response at the weaning stress. Furthermore, early weaning did not seem to affect liver functionality due to an overall increase in plasma albumin concentrations in both groups without differences [34]. Regarding liver function biomarkers, differences have been detected for GGT, involved in the metabolism of the amino acids, and ALP, which plays a role in dephosphorylating compounds. Their plasma increase could be seen as a proxy of liver damage [14] but our results showed that in all calves these parameters decreased, suggesting that weaning age did not affect the liver functionality in the post-weaning period. Pérez-Santos et al. [35] also reported a significant decrease in GGT activity consistently from birth up to 90 days of age and higher initial levels could be explained by the colostrum intake due to the higher concentration of GGT [5,36], implying that GGT absorbed from colostrum remain still elevated within 3three weeks of age. Hence, the greater activity at −25 days from weaning in EW calves is only due to their younger age (20 d vs. 35 d of age, respectively, for EW and CW) compared with CW calves.

The liver is also involved in PON activity regulation because it reduces albumin, PON, and lipoproteins during acute phase response [37,38]. PON is negative acute-phase protein, a marker of oxidative stress, and associated with blood high-density lipoproteins concentration [16]. In our study, PON was lower in EW calves, with also an increasing trend over time. In healthy neonates, the lower PON concentration during the first days of life may be explained by a lower metabolic activity of liver and lipid metabolism [39]. After weaning, both groups showed higher levels, but CW calves had the highest level that could be attributed to an older age, thus, ruling out any process associated with an inflammatory response due to weaning stress. The lower level of Ca in EW calves could have emphasized this event [40]. In line with PON, AOPP, a marker of protein oxidation and oxidative stress [41], showed the highest values in EW calves. The observed values were similar to those reported in other studies on dairy calves [42] but higher than those observed by Ranade et al. [43] on young ruminants.

The tendency to decrease over time may be linked to increased antioxidant defense [43]. In contrast with AOPP and PON, no differences on ROM were found between groups [44]. Weaning strategy effects involve mineral metabolism as calcium resulted to be dependent by Wa and Time only at 20 d with higher values in CW calves. The lower values for EW calves in the post-weaning period, at 20 d, could be explained by the lower starter intake. Nevertheless, the plasma calcium concentration was within the physiological range of adult bovine [45], so the reduced bone growth after EW would not be attributable with certainty to the lower calcium levels. In addition, the increasing concentration of Mg over time in both groups showed how rumen functions were not negatively affected but, rather, rumen correctly developed, as Mg is primely adsorbed by rumen epithelium.

Plasma biomarkers trends were compared according to days from weaning between calves’ groups, highlighting some significant differences, which in turn are related also with the physiological animal growth. The proposed results focused the aim that early weaning age did not affect the calves’ health status checked by plasma biomarkers among the main metabolism mechanisms.

## 5. Conclusions

The use of a proper weaning strategy for calves is fundamental to ensure optimal growth performance and development of the gastrointestinal tract and future performances. The present study evaluated the effects of early weaning (45 d) on growth and metabolic parameters in Simmental calves. The proposed results highlighted that early weaning might not affect inflammatory status and liver functionality after weaning. The average daily gain obtained by calves weaned at 45 d supported this strategy, despite the lower body weight at weaning and after (due only to the age difference of 15 days). Hence, early weaning in order to reduce rearing costs might not jeopardize calf development, as long as calves can reach body gains as reported in the present study. However, at least for performance, we are aware that the low number of calves enrolled in each treatment can limit the recommendations based on our findings. Thus, this study should be considered more like an explorative investigation since no recent data are available for Simmental calves in terms of growth performance and inflammometabolic adaptation.

## Figures and Tables

**Table 1:** Chemical composition of bulk whole milk and calf starter fed to calves.

Item	Bulk Whole Milk ^1^	Calf Starter ^2^	Grass Hay
N. of samples	13	1	1
% on as fed			
Fat	3.35	-	-
Protein	3.63	-	-
Lactose	4.67	-	-
Casein	2.79	-	-
True protein	3.43	-	-
Urea, mg/dL	30.38	-	-
Dry matter (DM), %	11.65	87.87	11.13
% on DM			
Starch	-	24.38	3.42
Crude Protein	-	18.59	7.64
Ether Extract	-	3.05	1.38
Neutral Detergent Fiber	-	33.22	65.51
Acid Detergent Fiber	-	23.18	37.66
Acid Detergent Lignin	-	6.85	5.5
Ash	-	7.34	9.78

^1^ Whole milk fed to calves collected from the tank, after each milking in the morning and in the evening. ^2^ Values are expressed on a DM basis.

**Table 2:** Plasma biomarkers of energy and protein metabolism (LSM) at pre-weaning (average between −25 and −15 d before weaning), weaning, and post-weaning period (average between +6 and +21 d after weaning) of Simmental calves weaned at 45 (EW) or 60 (CW) d of age.

Item ^1^	Pre-Weaning		Weaning		Post-Weaning		SEM ^2^	*p*-Value		
	EW	CW	EW	CW	EW	CW		Wa ^3^	Time ^4^	Wa × Time ^5^
Glucose, mmol/L	6.22	6.08	5.44	5.90	5.00	6.08	0.32	0.06	<0.05	<0.05
BHB, mmol/L	0.13	0.15	0.17	0.19	0.23	0.29	0.06	0.09	<0.05	0.92
NEFA, mmol/L	0.18	0.20	0.18	0.26	0.11	0.07	0.11	0.89	<0.05	0.88
Urea, mmol/L	3.24	3.62	3.71	4.07	4.04	4.20	0.58	0.50	0.20	0.85
Creatinine, mmol/L	113.06	116.41	121.20	115.05	110.97	108.44	12.22	0.94	0.15	0.65
Total protein, g/L	63.40	60.78	62.30	62.16	59.40	62.07	29.25	0.99	0.85	0.71

^1^ BHB = beta-hydroxybutyrate; NEFA = non-esterified fatty acids. ^2^ Greatest standard error of the mean. ^3^ Wa = overall effect of weaning age (45 vs 60 days of age). ^4^ Time = overall effect of days relative to weaning (−25, −15, 0, +6, and +21 d). ^5^ Wa × Time = interaction of weaning age and days relative to weaning time.

**Table 3:** Plasma biomarkers of oxidative status (LSM) at pre-weaning (average between −25 and −15 d before weaning), weaning, and post-weaning period (average between +6 and +21 d after weaning) of Simmental calves weaned at 45 (EW) or 60 (CW) d of age.

Item ^1^	Pre-Weaning		Weaning		Post-Weaning		SEM ^2^	*p*-Value		
	EW	CW	EW	CW	EW	CW		Wa ^3^	Time ^4^	Wa × Time ^5^
ROM, mg of H_2_O_2_/0.1 L	15.51	14.76	13.52	11.87	11.45	11.99	2.13	0.76	<0.05	0.54
MPO, U/L	249.73	262.69	173.16	282.48	297.68	296.05	92.61	0.59	0.63	0.83
AOPP, μmol/L	84.20	69.55	78.40	45.41	54.38	49.87	27.94	<0.05	0.12	0.58
FRAP, μmol/L	134.11	124.18	141.46	146.97	155.27	134.38	14.31	0.24	0.31	0.73

^1^ ROM = reactive oxygen metabolites; MPO = myeloperoxidase; AOPP = advanced oxidation protein products; FRAP = ferric-reducing ability of plasma. ^2^ Greatest standard error of the mean. ^3^ Wa = overall effect of weaning age (45 vs. 60 days of age). ^4^ Time = overall effect of days relative to weaning (−25, −15, 0, +6, and +21 d). ^5^ Wa × Time = interaction of weaning age and days relative to weaning time.

**Table 4:** Plasma parameters of liver metabolism and enzyme, inflammatory response, and minerals (LSM) at pre-weaning (average between −25 and −15 d before weaning), weaning, and post-weaning period (average between +6 and +21 d after weaning) of Simmental calves weaned at 45 (EW) or 60 (CW) d of age.

Item ^1^	Pre-Weaning		Weaning		Post-Weaning		SEM ^2^	*p*-Value		
	EW	CW	EW	CW	EW	CW		Wa ^3^	Time ^4^	Wa × Time ^5^
**Liver metabolism**										
Albumin, g/L	33.13	33.86	34.85	34.95	33.63	34.89	1.22	0.43	<0.05	0.43
Globulin, g/L	30.27	26.92	27.45	27.21	25.77	27.18	1.99	0.59	0.39	0.33
Cholesterol, mmol/L	4.47	3.95	3.71	3.47	2.17	2.44	0.43	0.70	<0.05	<0.05
Triglycerides, mg/dL	0.40	0.31	0.35	0.19	0.22	0.25	0.07	0.13	<0.05	0.34
PON, U/mL	61.11	97.40	89.31	117.32	86.35	113.18	12.40	<0.05	<0.05	0.43
**Inflammatory response**										
Haptoglobin, g/L	0.25	0.25	0.22	0.19	0.31	0.25	0.05	0.18	0.37	0.38
Ceruloplasmin, μmol/L	1.96	1.89	1.67	1.69	1.58	1.66	0.21	0.97	<0.05	0.74
**Minerals**										
Ca, mmol/L	2.79	2.74	2.65	2.74	2.53	2.70	0.11	0.32	<0.05	<0.05
Mg, mmol/L	0.86	0.88	0.96	0.96	0.90	0.98	0.05	0.23	<0.05	0.51
Zn, μmol/L	17.87	16.63	15.64	15.16	13.47	13.16	3.30	0.16	0.54	0.94
P, mmol/L	3.33	3.35	3.23	3.44	3.05	3.27	0.16	0.24	0.06	0.37
**Liver Enzymes**										
AST, U/L	54.18	71.47	64.82	97.27	83.37	126.42	31.90	0.11	0.41	0.90
GGT, U/L	110.92	47.69	34.07	25.34	20.05	19.72	27.14	0.29	<0.05	<0.05
ALP, U/L	421.02	535.87	370.21	413.17	310.45	417.49	104.65	0.32	<0.05	0.18
LH, U/L	1575.71	2020.83	1805.98	2294.98	1890.65	2626.99	451.05	0.16	<0.05	0.86

^1^ PON = paraoxonase; AST = aspartate aminotransferase; GGT = γ-glutamyl transferase; ALP = alkaline phosphatase; LH = lactate dehydrogenase. ^2^ Greatest standard error of the mean. ^3^ Wa = overall effect of weaning age (45 vs. 60 days of age). ^4^ Time = overall effect of days relative to weaning (−25, −15, 0, +6, and +21 d). ^5^ Wa × Time = interaction of weaning age and days relative to weaning time.

## Data Availability

Data available on request from the authors.
